# Supplementary figures and images for: Use of the Azure Kinect to measure foot clearance during obstacle crossing: A validation study
Source: PLoS One. 2022 Mar 11;17(3):e0265215. doi: 10.1371/journal.pone.0265215 (PMC8916621; doi:10.1371/journal.pone.0265215)

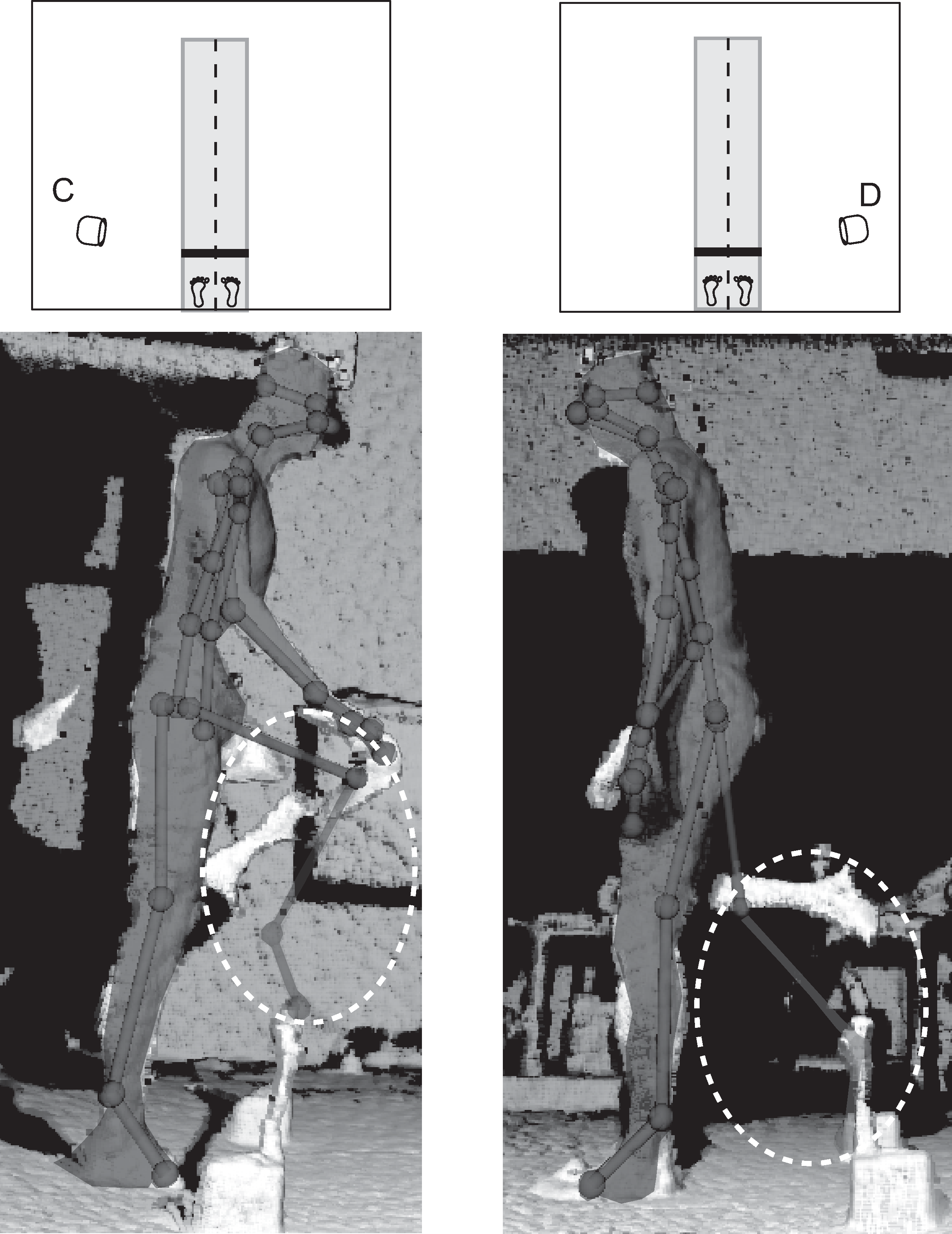

Supplement: S1 Fig — (TIF) [file pone.0265215.s001.tif]

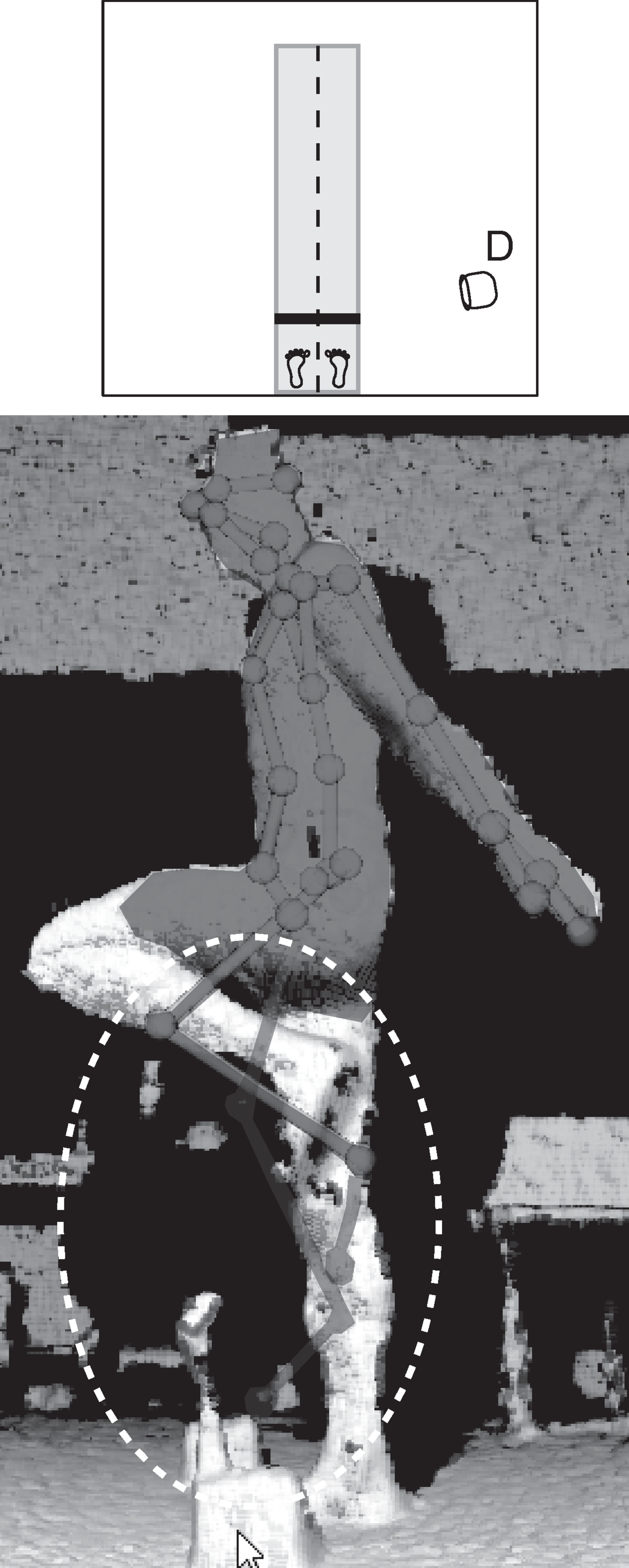

Supplement: S2 Fig — (TIF) [file pone.0265215.s002.tif]
