# Supplementary material for: Use of the Azure Kinect to measure foot clearance during obstacle crossing: A validation study
Source: PLoS One. 2022 Mar 11;17(3):e0265215. doi: 10.1371/journal.pone.0265215 (PMC8916621; doi:10.1371/journal.pone.0265215)
Supplement: Attachment — Submitted filename: Response to Reviewers.docx [file pone.0265215.s006.docx]

Dr. Shazlin Shaharudin

Editor-in-Chief, PLOS ONE

shazlin@usm.my

February 21, 2022

Ref.: PONE-D-21-33562

Use of the Azure Kinect to measure foot clearance during obstacle crossing: A validation study

Dear Dr. Shazlin Shaharudin

Thank you very much for editing our manuscript, “Use of the Azure Kinect to measure foot clearance during obstacle crossing: A validation study”, and the valuable comments of the two reviewers. We submit our revised manuscript, as well as a point-by-point response to the reviewers’ comments.

According to a comment from the academic editor of the journal, we have modified the statement of financial disclosure as follows. Also, we have added another funding source because we would pay the publishing fee from the grant (21H05334).

This study was supported by JSPS/MEXT KAKENHI Grant-in-Aid, Grant Number: 17H04750, 21H05334. The funders had no role in study design, data collection and analysis, decision to publish, or preparation of the manuscript.

We feel that the revised manuscript is a suitable response to the comment and is significantly improved over the initial submission.

Thank you in advance for your kind consideration of this paper.

Sincerely yours,

Masahiro SHINYA

Hiroshima University

1-7-1 Kagamiyama, Higashi-Hiroshima, 739-8521, Japan

+81-82-424-4544

[mshinya@hiroshima-u.ac.jp](mailto:mshinya@hiroshima-u.ac.jp)

**Academic editor:**

**1. Please ensure that your manuscript meets PLOS ONE's style requirements, including those for file naming. The PLOS ONE style templates can be found at**

**https://journals.plos.org/plosone/s/file?id=wjVg/PLOSOne_formatting_sample_main_body.pdf and**

[**https://journals.plos.org/plosone/s/file?id=ba62/PLOSOne_formatting_sample_title_authors_affiliations.pdf**](https://journals.plos.org/plosone/s/file?id=ba62/PLOSOne_formatting_sample_title_authors_affiliations.pdf)

We have confirmed that our manuscript meets PLOS ONE’s style requirement.

**2. Thank you for stating the following financial disclosure:**

**This study was supported by JSPS KAKENHI Grant Number 17H04750.**

**Please state what role the funders took in the study. If the funders had no role, please state: "The funders had no role in study design, data collection and analysis, decision to publish, or preparation of the manuscript."**

**If this statement is not correct you must amend it as needed.**

**Please include this amended Role of Funder statement in your cover letter; we will change the online submission form on your behalf.**

We will change our financial disclosure.

“This study was supported by JSPS/MEXT KAKENHI Grant-in-Aid, Grant Number: 17H04750, 21H05334. The funders had no role in study design, data collection and analysis, decision to publish, or preparation of the manuscript.”

**3. Please review your reference list to ensure that it is complete and correct. If you have cited papers that have been retracted, please include the rationale for doing so in the manuscript text, or remove these references and replace them with relevant current references. Any changes to the reference list should be mentioned in the rebuttal letter that accompanies your revised manuscript. If you need to cite a retracted article, indicate the article’s retracted status in the References list and also include a citation and full reference for the retraction notice.**

We have confirmed that our reference list is complete and correct.

**4. While revising your submission, please upload your figure files to the Preflight Analysis and Conversion Engine (PACE) digital diagnostic tool, https://pacev2.apexcovantage.com/. PACE helps ensure that figures meet PLOS requirements. To use PACE, you must first register as a user. Registration is free. Then, login and navigate to the UPLOAD tab, where you will find detailed instructions on how to use the tool. If you encounter any issues or have any questions when using PACE, please email PLOS at figures@plos.org. Please note that Supporting Information files do not need this step.**

We used PACE to check our figure files met the PLOS requirements, and revised the wasted space in Fig. 2 and Fig. 4.

**Reviewer #1:**

**The authors have reported data from kinect system reporting it's validity to capture foot clearance during obstacle navigation. Some of the minor concerns are as follows:**

Thank you very much for your review. We revised the manuscript according to your and the other reviewer’s comments. In the revised manuscript, the modification associated with your comments are highlighted in green, and those with the other reviewer are highlighted in blue. Comment-by-comment responses are as follows.

**Line 49: do you mean risk of fall while contact with obstacles? Also, contact with obstacles does not necessarily imply that the strategy has to be crossing the obstacles. Someone could just avoid crossing the obstacle and take a different route. So, overall, in the introduction, please build a strong argument regarding clinical or daily importance of obstacle crossing. Currently, it is lacking.**

We strengthened the Introduction by adding detailed description and references.

*“Tripping over obstacles is one of the most frequent cause of falls, reported that approximately 30 to 50 % of falls among elderly people were caused by tripping [2,3]. According to the systematic review by Galna et al. [4], research on effect of aging on obstacle-crossing behavior has been getting much attention.”* (line 43-46)

**Also, asymmetry in foot clearance is different than insufficient foot clearance which might be what you mean? So, please use the correct verbiage and reference in your context.**

**Line 51: You talk about variability which is a totally different biomechanical measure than asymmetry or clearance. May be you could put some more thought into the variables which are part of your manuscript and also related to the context of the introduction.**

As you mentioned, insufficient foot clearance might be related to an increased risk of tripping. However, the relevance of the foot clearance is not limited to its raw value, fall risk indicators might be obtained by processing foot clearance. Actually, researchers have calculated the asymmetry or variability in foot clearance and shown that these were related to the fall risk. To make this point clear, we added the following sentences.

*“The foot clearance is a versatile variable in fall prediction studies. Not only insufficient foot clearance (i.e., being short in absolute value), but large asymmetry or variability in foot clearance were also known as fall risk indicators.”* (line 49-51)

**Line 70: based on this, would not you propose just using the camera diagonally for your study if diagonally has already been shown to be better? Yes/no, and why needs to be added to your introduction section.**

To emphasize the differences between the previous and the present studies, we added the following description.

“*However, validation in the previous study was only conducted on the measurement of the unilateral kinematics because steady-state walking is a symmetrical movement.*” (line 72-74)

**Lines 72-78: seem thoughts have bene left incompletely addressed. Please reshape this paragraph.**

**Line 79: If the objective was foot clearance then why did you focus that you wanted to compare lead vs trail limb? And if you wanted to compare lead vs trail limb, then you need to state the reason and scientific and/or clinical reasoning for it.**

We have made a substantial revision on this paragraph. In this paragraph, we firstly state the importance of recording both sides in obstacle crossing tasks. Then, we point out the potential self-occlusion problem, and finally, we briefly explain the comparison in the present study.

*“Unlike the steady-state walking, researchers often record kinematics from the both of the lead and trail limbs. For example, the lower clearance in the trail limb compared to the lead limb was observed in older adults with cognitive impairment [5,8]. To calculate the asymmetry of the foot clearance, systematic errors should be identical for the both sides. If one tries to record the kinematics from both sides by using a single Kinect sensor, self-occlusion could drastically impact the measurement quality. Seo et al. [19] reported that the contralateral Kinect location made a large error due to being occluded by other body parts. This means that the measurement performance could be different between the left and right sides of the body if the motion was captured from the Kinect placed at one side of the participant. In this study, we compared the measurement performance between the lead and trail limbs during obstacle crossing.”* (line 75-83)

**Line 111: was there any familiarization trial? Yes/No and why? Pl add this information.**

*“The participants practiced up to 3 trials before the recording trials for each obstacle condition.”* (line 105)

**Line 166: how many these happened in your study?**

The numbers of participants analyzed (i.e., not-excluded participants) were shown in Table 1. We added the explanation in the top of the paragraph describing the errors in foot clearance.

*“If the number of measurement failures exceeded 5 out of 10 trials for each condition for a participant, the data of the participants were excluded from the subsequent analysis of foot clearance. For example, n = 3 for L250 (Table 1) means that 13 participants were excluded from the following analysis because of this criterion.”* (line 239-242)

**Reviewer #2:**

**Reviewer finds the article to be reasonably written.**

Thank you very much for your review. We revised the manuscript according to your and the other reviewer’s comments. In the revised manuscript, the modification associated with your comments are highlighted in blue, and those with the other reviewer are highlighted in green. Comment-by-comment responses are as follows.

**Line 204 . Suggest to reintroduce all the acronyms i.e. L50, T250 in results**

In the revised manuscript, the acronyms in the Methods section are removed, and they are introduced in the top of the Results section.

*“When we describing the results of the Friedman tests, we use abbreviations for mentioning the limb and the obstacle height conditions: the lead limb in the 50 mm obstacle condition (L50), the trail limb in the 250 mm obstacle condition (T250), etc.”* (line 209-211)

**Line 227 . Table 1; perhaps this table can be simplified or organised in a way that shows the main significance?**

To clarify the significance, we added the asterisks in the Table 1.

Because descriptive are important information for the readers, we would like to show the values in the manuscript. For those who plan to use Kinect in their study, the quantitative information (e.g., if the Kinect was placed diagonally (B), recording accuracy would be about 6.4 mm for the trail limb for a 50 mm obstacle) would be of great value than the quantitative information (e.g., Diagonal Kinect placement is better than the straight/sideway placements.).
